# Characteristics of spontaneous nystagmus and its correlation to video head impulse test findings in vestibular neuritis

**DOI:** 10.3389/fnins.2023.1243720

**Published:** 2023-08-22

**Authors:** Xueqing Zhang, Qiaomei Deng, Yao Liu, Shanshan Li, Chao Wen, Qiang Liu, Xiaobang Huang, Wei Wang, Taisheng Chen

**Affiliations:** ^1^Department of Otorhinolaryngology Head and Neck Surgery, Tianjin First Central Hospital, Tianjin, China; ^2^Institute of Otolaryngology of Tianjin, Tianjin, China; ^3^Key Laboratory of Auditory Speech and Balance Medicine, Tianjin, China; ^4^Key Medical Discipline of Tianjin (Otolaryngology), Tianjin, China; ^5^Quality Control Centre of Otolaryngology, Tianjin, China

**Keywords:** spontaneous nystagmus, vHIT, vestibular neuritis, semicircular canals, nystagmus direction

## Abstract

**Objective:**

To explore the direction and SPV (slow phase velocity) of the components of spontaneous nystagmus (SN) in patients with vestibular neuritis (VN) and the correlation between SN components and affected semicircular canals (SCCs). Additionally, we aimed to elucidate the role of directional features of peripheral SN in diagnosing acute vestibular syndrome.

**Materials and methods:**

A retrospective analysis was conducted on 38 patients diagnosed with VN in our hospital between 2022 and 2023. The direction and SPV of SN components recorded with three-dimensional videonystagmography (3D-VNG) and the video head impulse test (vHIT) gain of each SCC were analyzed as observational indicators. We examined the correlation between superior and inferior vestibular nerve damage and the direction and SPV of SN components, and vHIT gain values in VN patients.

**Results:**

The median illness duration of between symptom onset and moment of testing was 6 days among the 38 VN patients (17 right VN and 21 left VN). In total, 31 patients had superior vestibular neuritis (SVN), and 7 had total vestibular neuritis (TVN). Among the 38 VN patients, all had horizontal component with an SPV of (7.66 ± 5.37) °/s, 25 (65.8%) had vertical upward component with a SPV of (2.64 ± 1.63) °/s, and 26 (68.4%) had torsional component with a SPV of (4.40 ± 3.12) °/s. The vHIT results in the 38 VN patients showed that the angular vestibulo-ocular reflex (aVOR) gain of the anterior (A), lateral (L), and posterior (P) SCCs on the ipsilesional side were 0.60 ± 0.23, 0.44 ± 0.15 and 0.89 ± 0.19, respectively, while the gains on the opposite side were 0.95 ± 0.14, 0.91 ± 0.08, and 0.96 ± 0.11, respectively. There was a statistically significant difference in the aVOR gain between the A-, L-SCC on the ipsilesional side and the other SCCs (*p* < 0.001). The aVOR gains of A-, L-, and P-SCC on the ipsilesional sides in 31 SVN patients were 0.62 ± 0.24, 0.45 ± 0.16, and 0.96 ± 0.10, while the aVOR gains on the opposite side were 0.96 ± 0.13, 0.91 ± 0.06, and 0.98 ± 0.11, respectively. There was a statistically significant difference in the aVOR gain between the A-, L-SCC on the ipsilesional side and the other SCCs (*p* < 0.001). In 7 TVN patients, the aVOR gains of A-, L-, and P-SCC on the ipsilesional side were 0.50 ± 0.14, 0.38 ± 0.06, and 0.53 ± 0.07, while the aVOR gains on the opposite side were 0.93 ± 0.17, 0.90 ± 0.16, and 0.89 ± 0.09, respectively. There was a statistically significant difference in the aVOR gain between the A-, L-, and P-SCC on the ipsilesional side and the other SCCs (*p* < 0.001). The aVOR gain asymmetry of L-SCCs in 38 VN was 36.3%. The aVOR gain asymmetry between bilateral A-SCCs and bilateral P-SCCs for VN patients with and without a vertical upward component was 12.8% and 8.3%, which was statistically significant (*p* < 0.05). For VN patients with and without a torsional component, the aVOR gain asymmetry of bilateral vertical SCCs was 17.0% and 6.6%, which was statistically significant (*p* < 0.01). Further analysis revealed a significant positive correlation between the aVOR gain asymmetry of L-SCCs and the SPV of the horizontal component of SN in all VN patients (*r* = 0.484, *p* < 0.01), as well as between the asymmetry of bilateral vertical SCCs and the SPV of torsional component in 26 VN patients (*r* = 0.445, *p* < 0.05). However, there was no significant correlation between the aVOR gains asymmetry of bilateral A-SCCs and P-SCCs and the SPV of the vertical component in 25 VN patients.

**Conclusion:**

There is a correlation between the three-dimensional direction and SPV characteristics of SN and the aVOR gain of vHIT in VN patients. These direction characteristics can help assess different SCCs impairments in patients with unilateral vestibular diseases.

## 1. Introduction

Spontaneous nystagmus (SN) is a common clinical sign of peripheral vestibular disorders. SN is typically horizontal or horizontal-torsional, direction-fixed, and enhanced by removing visual fixation, and its SPV follows Alexander’s law (Liu et al., 2017). It is an objective indicator of asymmetric static tension in the bilateral vestibular system (Liu et al., 2017). Vestibular neuritis (VN) is a vestibular syndrome caused by acute unilateral vestibulopathy and is the second leading cause of peripheral vestibular vertigo: with the first being benign paroxysmal positioning vertigo (BPPV) (Le et al., 2019). There is unambiguous evidence of reduced aVOR function on the side opposite to the direction of the fast phase of the SN in VN (Aw et al., 2001; Baier et al., 2008; Bachmann et al., 2018). Unidirectional horizontal-torsional SN beats to the healthy side, and the SPV weakens with the establishment of vestibular compensation (Lacour et al., 2016). In 1996, Fetter and Dichgans (1996) studied the three-dimensional (3D) properties of aVOR in 16 VN patients using 3D magnetic search coil eye movement recording and quantified the dynamic asymmetries. A large body of research has shown that VN is not a complete unilateral vestibular lesion; instead, it most commonly affects only the upper branch of the vestibular nerve innervating the anterior (A)-semicircular canal (SCC), L-SCC, the utricle, and their afferents (Sando et al., 1972; Buki and Ward, 2021). Therefore, patients with unilateral VN show significantly reduced aVOR gain values of A- and L-SCCs on the ipsilesional side and present with covert or overt saccades (Psillas et al., 2022). This study aimed to investigate impairments in SCCs and explore the relationship between the direction characteristics of SN and various SCCs impairments. This was achieved by analyzing the 3D direction and SPV of SN in patients with unilateral VN and combining them with the aVOR gain and aVOR gain asymmetry in vHIT. This study’s findings will assist in further evaluating different SCCs impairments in patients with unilateral vestibular diseases.

## 2. Materials and methods

### 2.1. Participants

This retrospective study involved the assessment of 38 patients with unilateral VN or acute unilateral vestibulopathy (AUVP) patients, examined at the Ear, Nose, and Throat (ENT) Department of MY Hospital, Tianjin First Central Hospital between 2022 and 2023. Of 38 patients, 31 had superior vestibular neuritis (SVN), and seven had total vestibular neuritis (TVN). All the subjects provided informed consent to be included in the study. The study procedures were approved by the Ethics Committee of Tianjin First Central Hospital.

We included patients diagnosed with AUVP or VN according to the multidisciplinary experts’ consensus on vestibular neuritis (Professional Committee on Vertigo, 2020) and the diagnostic criteria of AUVP or VN (Strupp et al., 2022).

We exclude patients with unclear diagnoses or diagnostic controversies. Additionally, we excluded patients with coexistence of other diseases, such as BPPV, Meniere disease, sudden deafness with vertigo, Ramsay Hunt syndrome, labyrinthitis, as well as head trauma, vestibular migraine, stroke, and other central vestibular vertigo and balance disorders.

### 2.2. Study procedure

We obtained a detailed medical history of the onset of symptoms and their severity, illness duration, and associated factors. VN and AUVP were diagnosed with the results of SN, vHIT, and caloric test. The lesion side, superior or inferior vestibular nerve damage, and associated SCCs were determined. SN, vHIT, caloric test, and corresponding parameters were observed and recorded using 3D-VNG (VertiGoggles-M, ZEHNIT Medical Technology-VNG-II, Shanghai, China).

The VOR gain of vHIT is the ratio of eye velocity to head velocity, with a normal values range of 0.8–1.2 for L-SCCs and 0.7–1.2 for vertical SCCs. Unilateral weakness (UW) ≥25% and directional preponderance (DP) ≥30% indicate an abnormal caloric test.

### 2.3. Analysis

The main measures in vHIT are the aVOR gain, aVOR gain asymmetry, and the presence or absence of compensatory saccades. The aVOR gain assesses the function of the SCCs and their corresponding nerves on both sides. The aVOR gain asymmetry is a good sensitivity and specificity test for the early diagnosis of VN associated with acute vertigo. We analyzed the 3D direction and SPV (slow phase velocity) of SN and the aVOR gain of vHIT in patients with unilateral VN. The horizontal component of SN is caused by the imbalance of aVOR in the bilateral L-SCCs, while the vertical and torsional components are the comprehensive vectors of bilateral A- and P-SCCs effects.

The formula for calculating the aVOR gain asymmetry of vHIT is as follows:

1. The aVOR gain asymmetry of L-SCCs:

$$|\left(L_{\mathrm{ipsilesional}}-L_{\mathrm{opposite}}\right)/\left(L_{\mathrm{ipsilesional}}+L_{\mathrm{opposite}}\right)|*100\%$$

1. The aVOR gain asymmetry between bilateral A-SCCs and bilateral P-SCCs:

$$\begin{array}{l} |\left[\left(A_{\mathrm{ipsilesional}}+A_{\mathrm{opposite}}\right)-\left(P_{\mathrm{ipsilesional}}+P_{\mathrm{opposite}}\right)\right]\\ /\left[A_{\mathrm{ipsilesional}}+A_{\mathrm{opposite}}+P_{\mathrm{ipsilesional}}+P_{\mathrm{opposite}}\right]|*100\% \end{array}$$

1. The aVOR gains asymmetry of bilateral vertical SCCs:

$$\begin{array}{l} |\left[\left(A_{\mathrm{ipsilesional}}+P_{\mathrm{ipsilesional}}\right)-\left(A_{\mathrm{opposite}}+P_{\mathrm{opposite}}\right)\right]\\ /\left[A_{\mathrm{ipsilesional}}+P_{\mathrm{ipsilesional}}+A_{\mathrm{opposite}}+P_{\mathrm{opposite}}\right]|*100\% \end{array}$$

IBM SPSS Statistics 22 (IBM SPSS, Turkey) and JASP 0.16.3 (JASP, Netherlands) were used for statistical analyses. The quantitative data are presented as mean ± SD values and plotted using GraphPad Prism version 5 (GraphPad, San Diego, CA, United States). The strength of correlation (*r*) was calculated, and a *p*-value <0.05 was considered statistically significant.

## 3. Results

### 3.1. General demographic characteristics of subjects

**Figure 1:**
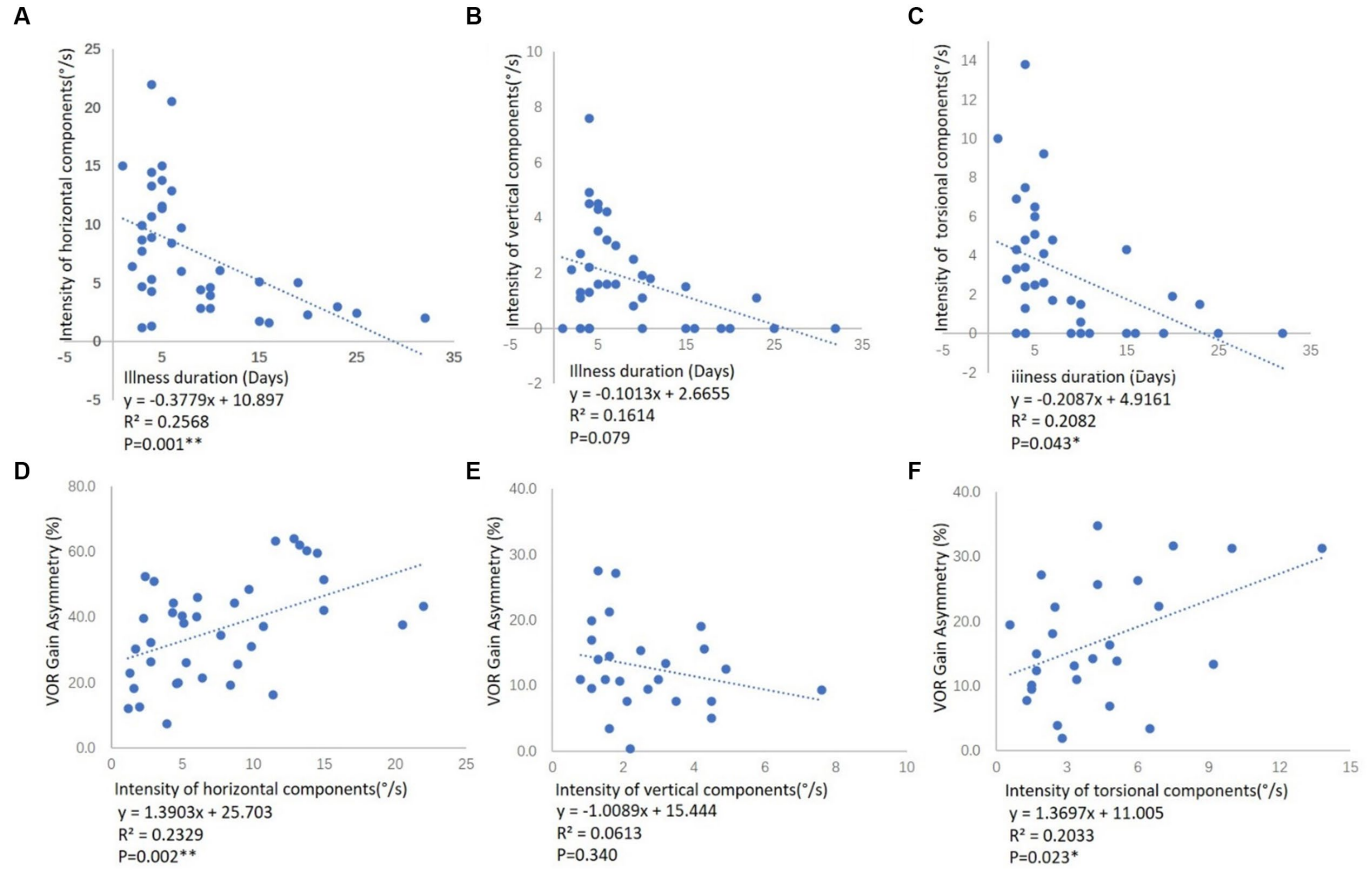
Correlation between the illness duration and the SPV of SN components in VN patients, and correlation between the SPV of SN components and aVOR gain asymmetry of vHIT in VN patients. **(A)** The correlation between the illness duration and the SPV of the horizontal component among all the VN patients. **(B)** The correlation between the illness duration and the SPV of the vertical component among 25 VN patients. **(C)** The correlation between the illness duration and the SPV of a torsional component among 26 VN patients. Illness duration means the time between symptom onset and moment of testing. The illness duration was negatively correlated with the horizontal and torsional components, respectively, but not with the vertical components. **(D)** The correlation between the aVOR gain asymmetry of L-SCCs and the SPV of the horizontal component among all the VN patients. **(E)** The correlation between the aVOR gain asymmetry of bilateral A- and P-SCCs and the SPV of the vertical component among 25 VN patients. **(F)** The correlation between the aVOR gain asymmetry of bilateral vertical SCCs and the SPV of a torsional component among 26 VN patients. *R*^2^ represents the goodness of fit, and *p* < 0.05 indicates a significant correlation between aVOR gain asymmetry and the SPV of SN components.

The ages of the 38 patients with VN (26 male and 12 female) ranged from 18–68 years (mean 42.21). Seventeen of the 38 patients had right-VN (13 male and 4 female) and their ages ranged from 20–62 years (mean 42.76), while 21 patients had left-VN (13 male and 8 female) with age ranges from 18–68 years (mean 41.76). Thirty one patients had superior-VN (22 male and 9 female), and their ages ranged from 18–68 years (mean 40.68); 7 patients had total-VN (4 male and 3 female) with ages ranging from 43–62 years (mean 49.00). Demographic data for VN are shown in Table 1. There were no significant differences in age or sex ratio between groups (*p* > 0.05). All VN patients had a median illness duration of between symptom onset and moment of testing of 6 days. And the illness duration was negatively correlated with the horizontal and torsional components, respectively, but not with the vertical components (Figures 1A–C).

**Table 1:** Demographic features of subjects in the vestibular neuritis groups.

Group feature	R-VN	L-VN	SVN	TVN	VN
Number	17	21	31	7	38
Age (years)^*^	42.76 ± 13.25	41.76 ± 14.11	40.68 ± 14.05	49.00 ± 8.93	42.21 ± 13.55
Sex (M:F)^*^	13:4	13:8	22:9	4:3	26:12

VN, vestibular neuritis; RVN, right VN; LVN, left VN; SVN, superior VN; TVN, total VN; M, male; F, female; ^*^p > 0.05.

### 3.2. Direction and SPV of spontaneous nystagmus of VN

The 3D direction and SPV of SN in the 38 VN patients were recorded and analyzed. The direction of the horizontal component was toward the opposite side, and the SPV was 7.66 ± 5.37°/s. About 65.7% (25/38) of the patients had a vertical upward component with a SPV of 2.64 ± 1.63°/s, and 68.4% (26/38) had a torsional component with a SPV of 4.40 ± 3.12°/s. Among the 17 RVN patients, all cases had a left horizontal component with a SPV of 11.30 ± 5.42°/s, 12 cases had a vertical upward component with a SPV of 3.47 ± 1.78°/s, and 13 cases had a torsional component with the direction of upper pole of the eye beating toward the right ear (from patients’ perspective) with a SPV of 5.46 ± 3.72°/s. Among the 21 LVN cases, 21 had a right horizontal component with a SPV of 4.70 ± 3.05°/s, 13 cases had a vertical upward component with a SPV of 1.87 ± 1.02°/s, and 13 cases had a torsional component with the direction of upper pole of the eye beating toward the left ear (from patients’ perspective) with a SPV of 3.35 ± 2.01°/s. Of the 31 SVN patients, the SPV of the horizontal component was 7.38 ± 5.49°/s, 23 cases had a vertical upward component with a SPV of 2.64 ± 1.67°/s, and 19 cases had a torsional component with a SPV of 4.41 ± 3.19°/s. In the 7 TVN patients, all cases had a horizontal and torsional components with SPV of 8.87 ± 4.98°/s and 5.54 ± 2.61°/s respectively, and 2 case had vertical component with SPV of 2.50 ± 1.41°/s (Table 2).

**Table 2:** The direction and SPV of spontaneous nystagmus in 38 VN patients.

Comp.	Direction and SPV	Direction	R-VN (17 cases)	L-VN (21 cases)	SVN (31cases)	TVN (7 cases)	VN (38 cases)
H	Direction	Left	17	0	13	4	17
		Right	0	21	18	3	21
		/	0	0	0	0	0
	SPV (°/s)		11.30 ± 5.42	4.70 ± 3.05	7.38 ± 5.49	8.87 ± 4.98	7.66 ± 5.37
V	Direction	Upward	12	13	23	2	25
		Downward	0	0	0	0	0
		/	5	8	8	5	13
	SPV (°/s)		3.47 ± 1.78	1.87 ± 1.02	2.64 ± 1.67	2.50 ± 1.41	2.64 ± 1.63
T	Direction	Right	13	0	9	4	13
		Left	0	13	10	3	13
		/	4	8	12	0	12
	SPV (°/s)		5.46 ± 3.72	3.35 ± 2.01	4.41 ± 3.19	5.54 ± 2.61	4.40 ± 3.12

Comp., component; H, horizontal component; V, vertical component; T, torsional component; left/right, upward/downward, right/left (upper pole of the eye beating toward the right/left ear from patients’ perspective), the direction of a component; /, no nystagmus.

### 3.3. Characteristics of vHIT and caloric test in VN

**Figure 2:**
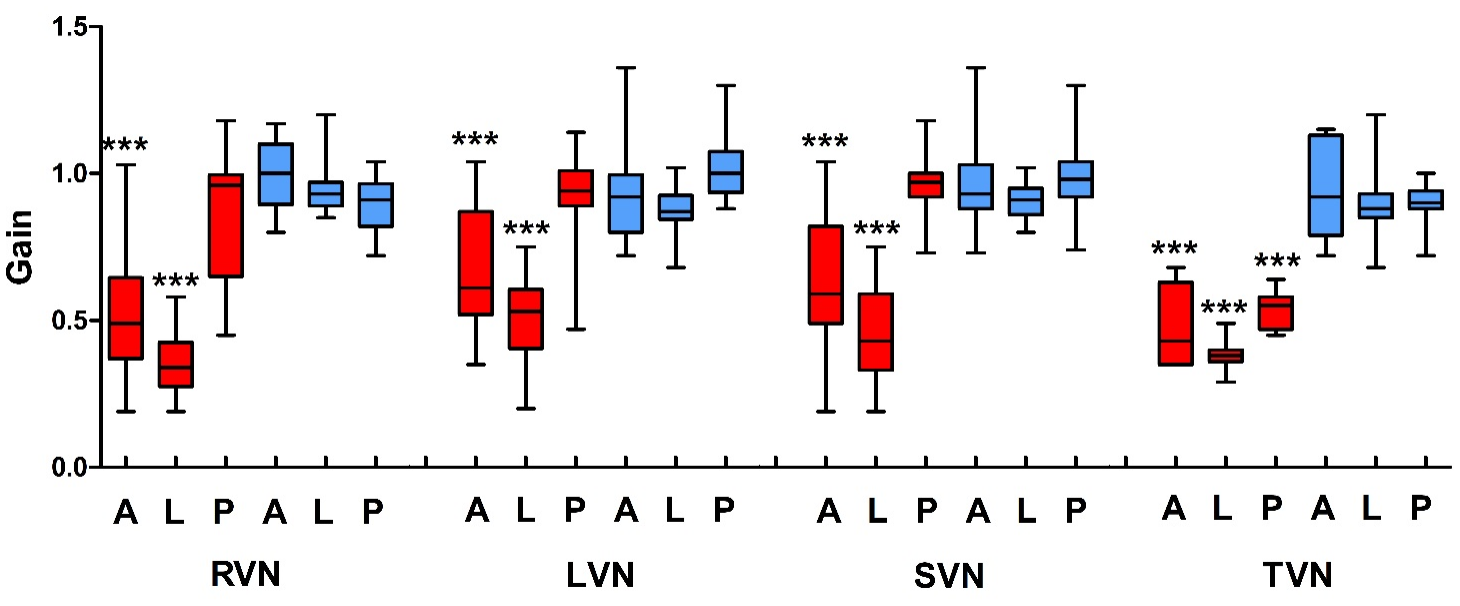
The aVOR gain of vHIT in vestibular neuritis patients. VN, vestibular neuritis; RVN, right VN; LVN, left VN; SVN, superior VN; TVN, total VN; A, anterior; L, lateral; P, posterior; red, the right semicircular canals; blue, left semicircular canals.

The aVOR gain and aVOR gain asymmetry of vHIT were analyzed in all 38 VN cases. The aVOR gains of the A-, L- and P-SCCs on the ipsilesional side were 0.60 ± 0.23, 0.44 ± 0.15, 0.89 ± 0.19, and those on the opposite side were 0.95 ± 0.14, 0.91 ± 0.08, 0.96 ± 0.11, respectively. There were significant differences between the gains of the A- and L-SCCs on the ipsilesional side and those of the other SCCs (*p* < 0.001). In the 17 RVN patients, the gains of A-, L-, and P-SCCs on the right side were 0.51 ± 0.21, 0.35 ± 0.11, and 0.85 ± 0.21, and those on the left side were 0.99 ± 0.12, 0.94 ± 0.08, and 0.89 ± 0.09, respectively. There were significant differences between the gains of the A- and L-SCCs on the right side and those of the other SCCs (*p* < 0.001). In the 21 LVN patients, the gains of A-, L-, and P-SCCs on the right side were 0.92 ± 0.14, 0.88 ± 0.07, and 1.02 ± 0.10, and those on the left side were 0.67 ± 0.22, 0.51 ± 0.15, and 0.91 ± 0.17, respectively. There were significant differences between the gains of the A- and L-SCCs on the left side and those of the other SCCs (*p* < 0.001). In the 31 SVN patients, the gains of A-, L-, and P-SCCs on the ipsilesional side were 0.62 ± 0.24, 0.45 ± 0.16, 0.96 ± 0.10, and those on the opposite were 0.96 ± 0.13, 0.91 ± 0.06, and 0.98 ± 0.11, respectively. There were significant differences between the gains of the A- and L-SCCs on the ipsilesional side and those of the other SCCs (*p* < 0.001). Among the 7 TVN patients, the gains of A-, L-, and P-SCCs on the ipsilesional side were 0.50 ± 0.14, 0.38 ± 0.06, and 0.53 ± 0.07, and those on the opposite were 0.93 ± 0.17, 0.90 ± 0.16, and 0.89 ± 0.09, respectively. There were significant differences between the gains of the A-, L-, and P-SCCs on the ipsilesional side and those of the other SCCs (*p* < 0.001) (Figure 2).

The caloric test showed decreased aVOR function of L-SCCs on the ipsilesional side at low frequency (0.003 Hz) in all the VN patients and DP to the opposite side (UW = 58.51 ± 22.89 > 25, DP = 83.37 ± 25.65 > 30). The UW and DP were (47.60 ± 20.69) and (89.00 ± 27.11) in the 17 RVN patients and were (66.70 ± 22.02) and (79.15 ± 25.04) in the 21 LVN patients, respectively, consistent with previous research results (Molnar et al., 2023).

### 3.4. Correlation between SN and vHIT gain in VN

**Figure 3:**
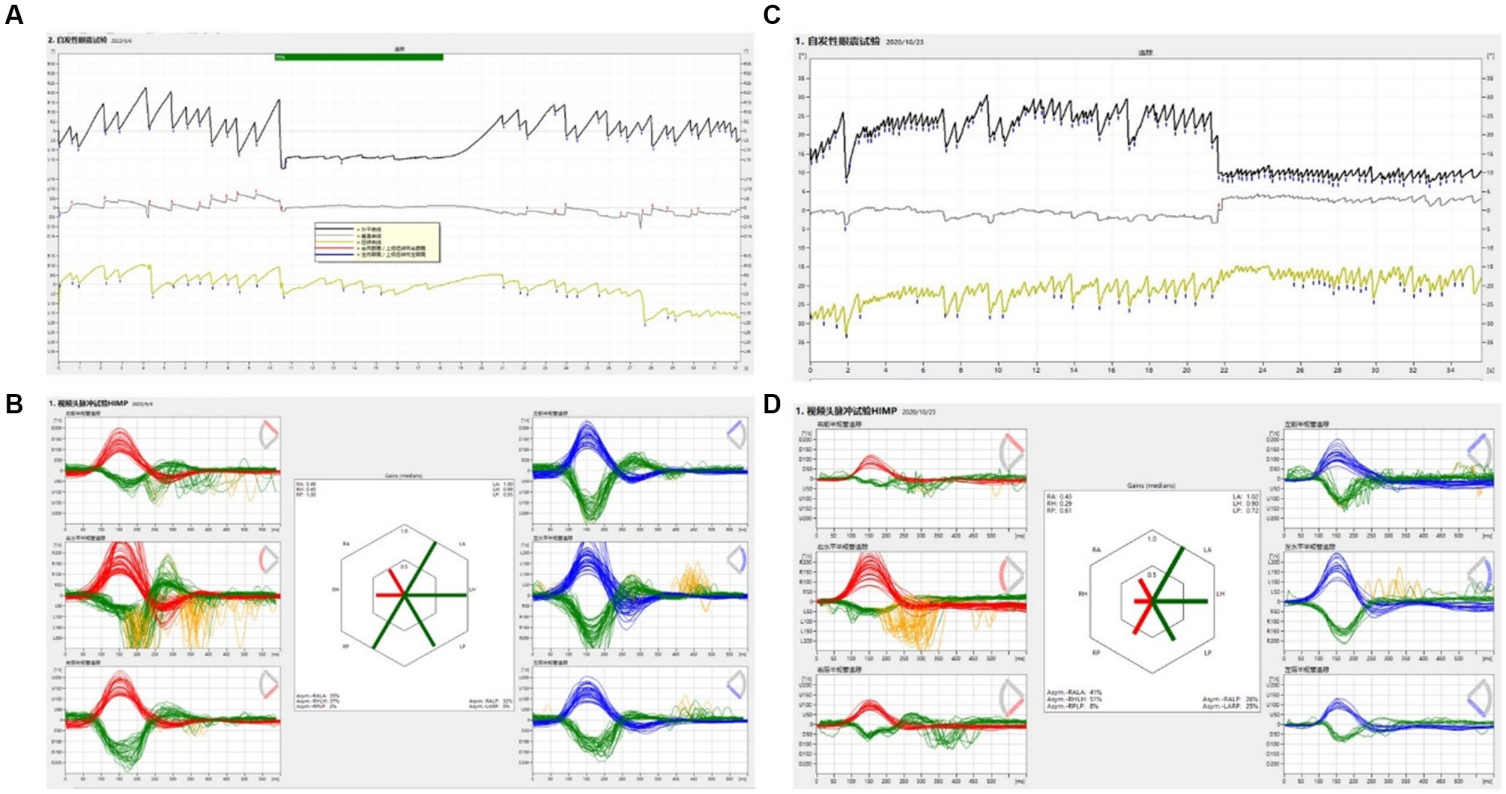
The SN and vHIT in SVN and TVN patients. **(A)** The SN of a SVN patient. Left horizontal (black), upward vertical (gray), and torsional (yellow) components. Nystagmus decreased after fixation. **(B)** vHIT of a SVN patient. Low aVOR gain in the A- and L-SCC on the right side with overt and covert saccades, and normal gain and no saccade in other SCCs. **(C)** The SN of a TVN patient. Left horizontal (black), no vertical (gray), and torsional (yellow) components. Nystagmus decreased after fixation. **(D)** vHIT of a TVN patient. Low aVOR gains in both the A-, L- and P-SCCs on the right side, accompanied by overt and covert saccades, and normal gain and no saccade in Left SCCs.

All patients with VN had SN directed toward the opposite side (Figure 3). We analyzed the aVOR gain asymmetry of different SCCs in vHIT. The aVOR gain asymmetry of L-SCCs in 38 VN was 36.3%. For VN patients with a vertical upward component (65.8%, 25/38), the aVOR gain asymmetry between bilateral A-SCCs and bilateral P-SCCs was 12.8%, while the asymmetry for those without a vertical component was 8.3%, and these differences were statistically significant (*p* < 0.05). For VN patients with a torsional component (68.4%, 26/38), the aVOR gain asymmetry of bilateral vertical SCCs was 17.0%, while the asymmetry for those without a vertical component was 6.6%, and these differences were statistically significant (*p* < 0.01).

We further analyzed the correlation between the aVOR gain asymmetry of SCCs in vHIT and the SPV of 3D components of SN in VN patients (Figures 1D–F). There was a significant positive correlation between the aVOR gain asymmetry of L-SCCs and the SPV of the horizontal component among all the VN patients (*r* = 0.484, *p* < 0.01), as well as the asymmetry of bilateral vertical SCCs and the SPV of torsional component in 26 VN patients (*r* = 0.445, *p* < 0.05). However, no significant correlations were found in asymmetry of bilateral A- and P-SCCs and the SPV of vertical component among 25 VN patients.

## 4. Discussion

Spontaneous nystagmus (SN)—present in a static state with the head upright and looking straight forward without any inducing condition—is common in acute unilateral vestibular dysfunction, which is spontaneous, unilateral and caused by asymmetric hypo-function of the angular vestibulo-ocular reflex (aVOR) (Wenyu et al., 2017; Ling et al., 2023). In the diagnosis, treatment, and rehabilitation of vestibular diseases, SN has the following clinical significance as a routine physical examination indication: (1) it identifies a central or peripheral disease based on the fixation inhibition test and changes in the direction and SPV of nystagmus during left and right fixation (Alexander’s law); (2) it indicates the side of the peripheral vestibular lesion. The direction of the SN points toward the higher tension side of the bilateral vestibule, which is usually the healthy side. For example, a right SN indicates a left peripheral vestibular lesion; (3) it evaluates the status of compensation. Patients with SN often indicate that vestibular static compensation has not been established; (4) it affects other vestibular examination results. SN of a certain SPV often affects the results of visual eye movement tests and caloric tests (Liu et al., 2017; Xie et al., 2021); (5) based on the theory of vestibular frequency, SN is a common sign of vestibular lesion at various frequencies (Deng et al., 2023); and (6) it traces the lesion of SCCs. Vestibular SN is a sign of SCCs pathway damage, but its relationship with SCCs lesions is not fully understood.

Ewald’s law, derived from animal experiments, reveals the physiological effects of endolymph flowing in a single SCC, including the plane, direction, and SPV of nystagmus. One of its main connotations is that the direction of nystagmus is the same as the plane of the stimulated SCCs. The law helps in understanding the physiological and pathological characteristics of human SCCs. Nystagmus induced by unilateral L-SCC stimulation was mainly the horizontal component accompanied by a weak vertical upward component. By contrast, nystagmus induced by unilateral P-SCC stimulation was chiefly vertical, upward, and torsional, accompanied by a weak horizontal component. Meanwhile, nystagmus induced by unilateral A-SCC stimulation was vertical, downward, and torsional nystagmus (Eggers et al., 2019). The nystagmus induced by BPPV follows the physiological effects of endolymph flowing in a single SCC. Previous studies have shown that the characteristics of nystagmus in horizontal semicircular canal canalolithiasis (HSC-Can) (Zhang et al., 2021, 2022) and posterior semicircular canal canalolithiasis (PSC-Can) (Liu et al., 2022) align with Ewald’s law and can be used as excellent physiological stimulation model of SCCs in humans.

However, peripheral vestibular diseases represented by VN in clinical often involve two or more SCCs pathways, resulting in a combined vector feature of multiple SCCs effects in the direction of SN. This interplay makes it challenging for clinicians to observe and understand SN and its intrinsic pathology. 3D-VNG technology can help analyze the combined vector features of SN. According to Ewald’s law, analyzing the characteristics of SN components using 3D-VNG technology and tracing the damage to the affected SCCs pathway is a new perspective for the clinical diagnosis of vestibular disease. Our study focuses on patients with a confirmed diagnosis of VN or AUVP and combines vHIT with 3D-VNG technology to explore the 3D characteristics of nystagmus after different SCCs aVOR injuries.

VN is a unilateral vestibular lesion mainly affecting the superior division of the vestibular nerve (Chang et al., 2022; Paris et al., 2022)—injuries to both the superior and inferior vestibular nerves are rare, whereas the inferior vestibular nerve is most often spared (Lee et al., 2019). Studies have shown that SN in SVN is horizontal, vertical, and torsional, whereby L-SCC afferent nerve dysfunction leads to the horizontal component, while A-SCC afferent nerve dysfunction leads to the torsional and weak vertical upward components (Fetter and Dichgans, 1996; Yagi et al., 2010). In the current study, the results of the vHIT and the caloric test demonstrated that the superior vestibular nerve was affected in 31 of the 38 VN patients, while both the superior and inferior vestibular nerves were affected in 7 of the 38 patients. The median illness duration of between symptom onset and moment of testing was 6 days. Static compensation had not yet been established, and SN was present in all patients. And the illness duration was negatively correlated with the horizontal and torsional components, respectively, but not with the vertical components (Figures 1A–C). All 38 VN patients in this study had a horizontal component toward the opposite side, 65.8% (25/38) had a vertical upward component, 68.4% (26/38) had a torsional component, and no patient had a vertical downward component. The results of vHIT showed significantly reduced aVOR gain values on the ipsilesional L-SCC with normal aVOR gain in the opposite L-SCC, and the asymmetry of L-SCCs was 36.3%. For VN patients with a vertical upward component, the aVOR gain asymmetry between bilateral A-SCCs and bilateral P-SCCs was 12.8%, while the asymmetry was 8.3% for those without a vertical component, and these differences were statistically significant (*p* < 0.05). And for VN patients with a torsional component, the aVOR gains asymmetry of bilateral vertical SCCs was 17.0%, while the asymmetry was 6.6% for those without a vertical component, and these differences were statistically significant (*p* < 0.01).

Further analysis of the correlation between the aVOR gain asymmetry and the SPV of SN components revealed a significant positive correlation between the aVOR gain asymmetry of L-SCCs and the SPV of horizontal component among the 38 VN patients (*r* = 0.484, *p* < 0.01), as well as between the asymmetry of bilateral vertical SCCs (analysis in Materials and methods) and the SPV of torsional component in 26 VN patients (*r* = 0.445, *p* < 0.05), while there was no significant correlations in asymmetry of bilateral A- and P-SCCs and the SPV of vertical component. However, no effective correlation analysis could be conducted due to the small sample size. Additionally, the intensities of SN components induced by the A- and P-SCCs were 30% and 10% of that of the L-SCCs, respectively (Aw et al., 1998): the vertical components have low weights in nystagmus such that weak nystagmus values cannot show a significant correlation with vHIT gain.

In this study, patients with SVN had significantly lower vHIT gains of the A- and L-SCCs on the ipsilesional side than other SCCs. The aVOR of the L-SCC on the ipsilesional side was lower than that on the opposite side, and the aVOR of the A-SCC on the ipsilesional side was lower than that of the other vertical SCCs. The direction characteristics of SN included a horizontal component toward the opposite side, a vertical upward component, and a torsional component (upper pole of the eye beating toward the right ear in RVN and upper pole of the eye beating toward the left ear in LVN). In patients with TVN, the vertical components of the A- and P-SCCs cancel or partially cancel each other, while the torsional components are added up with a stronger SPV than that of SVN (Table 2), so that the SN appears as a horizontal-torsional component. Patients with IVN were not involved in this study. According to the results of this study and Ewald’s law, the afferent nerve of the affected P-SCC is dysfunctional, the aVOR of the affected P-SCC is lower than that of other vertical SCCs, and the direction of SN is a vertical downward with torsional component (upper pole of the eye beating toward the right ear in RVN and upper pole of the eye beating toward the left ear in LVN).

Among the VN patients included in this study, the superior vestibular nerve was affected in 31, while both the superior and inferior vestibular nerves were affected in 7. The number of cases was relatively small, and there was a lack of patients with a dysfunctional inferior vestibular nerve. It is impossible to describe and analyze the SN characteristics of a single P-SCC lesion. Therefore, the SN characteristics of a single P-SCC lesion and its correlation with VHIT gain need further study. In addition, the lack of VEMPs results makes it impossible to evaluate the impact of otolith damage on SN in VN patients. In contrast to vHIT and caloric testing, VEMPs are much less relevant for the diagnosis of VN/AUVP (Fife et al., 2017; Strupp et al., 2022), while it is necessary to improve the evaluation of otolith damage on SN in VN patients in the future.

In conclusion, we analyzed the correlation between the direction and SPV of SN components and vHIT gain. The results showed that the SPV of the SN horizontal components and torsional components in VN patients were positively correlated with the aVOR gain asymmetry of vHIT, and the direction of SN corresponded to the plane of the excitable SCCs. There is a horizontal-vertical upward-torsional nystagmus in SVN, while horizontal nystagmus with a strong torsional component is found with no vertical component in TVN (upper pole of the eye beating toward the right ear in RVN, upper pole of the eye beating toward the left ear in LVN). Combining Ewald’s law and observing the 3D direction of SN in patients with VN/AUVP, it is of great clinical significance to locate the damaged SCCs and trace the target of the lesion. This study provides an objective basis for investigating the relationship between the SN direction and SCC lesions. It also clarifies the significance of the directional characteristics of vestibular peripheral SN components in diagnosing acute vestibular syndrome through medical history and examination.

## Data availability statement

The original contributions presented in the study are included in the article/Supplementary material, further inquiries can be directed to the corresponding author/s.

## Author contributions

WW and TC performed the study design. XZ and QD acquired and analyzed the data. XZ and TC drafted the manuscript. YL, SL, QL, CW, and XH revised the manuscript. All authors read and approved the final manuscript.

## Funding

This study was supported by the Tianjin Key Medical Discipline Construction Project (TJYXZDXK-046A), Tianjin Applied Basic Research Multiple Investment Fund Project (21JCQNJC01780 and 21JCQNJC01670), and Tianjin Health Research Project (TJWJ2022QN027, TJWJ2022QN028, and TJSJMYXYC-D2-021).

## Conflict of interest

The authors declare that the research was conducted in the absence of any commercial or financial relationships that could be construed as a potential conflict of interest.

## Publisher’s note

All claims expressed in this article are solely those of the authors and do not necessarily represent those of their affiliated organizations, or those of the publisher, the editors and the reviewers. Any product that may be evaluated in this article, or claim that may be made by its manufacturer, is not guaranteed or endorsed by the publisher.
